# Association of Trajectory of Cardiovascular Health Score and Incident Cardiovascular Disease

**DOI:** 10.1001/jamanetworkopen.2019.4758

**Published:** 2019-05-31

**Authors:** Shouling Wu, Shasha An, Weijuan Li, Alice H. Lichtenstein, Jingsheng Gao, Penny M. Kris-Etherton, Yuntao Wu, Cheng Jin, Shue Huang, Frank B. Hu, Xiang Gao

**Affiliations:** 1 Department of Cardiology, Kailuan General Hospital, Tangshan, People’s Republic of China; 2 Department of Emergency, HanDan Central Hospital, HanDan, People's Republic of China; 3 Vanderbilt University Medical Center/Vanderbilt Heart and Vascular Institute, Nashville, Tennessee; 4 Cardiovascular Nutrition Laboratory, Jean Mayer United States Department of Agriculture Human Nutrition Research Center on Aging, Human Nutrition Research Center on Aging, Tufts University, Boston, Massachusetts; 5 Department of Nutritional Sciences, Pennsylvania State University, State College; 6 Departments of Nutrition and Epidemiology, Harvard T.H. Chan School of Public Health, Boston, Massachusetts

## Abstract

**Importance:**

The American Heart Association 2020 Strategic Impact Goals target an improvement in overall cardiovascular health, as assessed by 7 health metrics (smoking, body weight, physical activity, diet, plasma glucose level, plasma cholesterol level, and blood pressure).

**Objective:**

To examine whether trajectories of overall cardiovascular health over time, as assessed by the cardiovascular health score (CHS) in 2006, 2008, and 2010, are associated with subsequent risk of CVD.

**Design, Setting, and Participants:**

The Kailuan study is a prospective, population-based study that began in 2006. The cohort included 74 701 Chinese adults free of myocardial infarction, stroke, and cancer in or before 2010. In the present study, CHS trajectories were developed from 2006 to 2010 to predict CVD risk from 2010 to 2015. Data analysis was performed from January 1, 2006, to December 31, 2015.

**Exposures:**

The CHS trajectories during 2006-2010 were identified using latent mixture models.

**Main Outcomes and Measures:**

Incident CVD events (myocardial infarction and stroke) during 2010-2015 were confirmed by review of medical records. The CHS trajectories were determined using 7 cardiovascular health metrics scored as poor (0 points), intermediate (1 point), and ideal (2 points); total score ranges from 0 (worst) to 14 (best). Based on the baseline CHS and patterns over time, 5 trajectories were categorized (low-stable, moderate-increasing, moderate-decreasing, high-stable I, and high-stable II).

**Results:**

Of the 74 701 adults included in the study (mean [SD] age at baseline, 49.6 [11.8] years), 58 216 (77.9%) were men and 16 485 (22.1%) were women. Five CHS trajectories were identified from 2006 to 2010: low-stable (n = 4393; range, 4.6-5.2), moderate-increasing (n = 4643; mean increase from 5.4 to 7.8), moderate-decreasing (n = 14 853; mean decrease from 7.4 to 6.3), high-stable I (n = 36 352; range, 8.8-9.0), and high-stable II (n = 14 461; range, 10.9-11.0). During 5 years of follow-up, 1852 incident CVD cases were identified. Relative to the low-stable trajectory, the high-stable II trajectory was associated with a lower subsequent risk of CVD (adjusted hazard ratio, 0.21; 95% CI, 0.16-0.26, after adjusting for age, sex, educational level, income, occupation, alcohol intake, and serum high-sensitivity C-reactive protein concentration at baseline).

**Conclusions and Relevance:**

Cardiovascular health trajectories may be associated with subsequent CVD risk.

## Key Points

**Question:**

Are trajectories of overall cardiovascular health over time, as assessed by the cardiovascular health score repeatedly in 2006, 2008, and 2010, associated with subsequent risk of cardiovascular disease?

**Findings:**

In this population-based study of 74 701 Chinese adults, 5 cardiovascular health score trajectories were identified. Relative to the lowest measured trajectory, the highest measured trajectory was associated with a 79% lower subsequent risk of cardiovascular disease after adjusting for age, sex, educational level, income, occupation, alcohol intake, and serum high-sensitivity C-reactive protein concentrations at baseline.

**Meaning:**

Long-term cardiovascular health trajectories may be associated with subsequent cardiovascular disease morbidity.

## Teaser

This population-based study examines changes in cardiovascular health status assessed by the Cardiovascular Health Score of Chinese adults over 5 years.

## Introduction

In 2010, the American Heart Association published its 2020 Strategic Impact Goals to improve the cardiovascular health of all Americans and changed the focus from primary prevention of cardiovascular disease (CVD) to primordial prevention.^1^ The committee proposed a new concept of ideal cardiovascular health, defined as the presence of 7 favorable health metrics and the absence of clinical CVD (eg, coronary heart disease and stroke).^1^ The 7 health metrics include 4 health behaviors (smoking, body weight, physical activity, and diet) and 3 health factors (blood pressure, plasma glucose level, and cholesterol level).^1^ Built on this concept and intended to capture individual-level changes in cardiovascular health factors and behaviors, a cardiovascular health score (CHS) was created based on the individual-level composite of all 7 cardiovascular health metrics to evaluate overall cardiovascular health status.^2^

The American Heart Association 2020 Strategic Impact Goals were conceptualized on the basis of effectiveness of primordial prevention, life-course nature of CVD development and risk factors, and balance between population- and individual-level prevention.^1,2^ The primordial prevention—the key component of promoting ideal cardiovascular health—focuses on preventing the initial development of risk factors by adopting healthier lifestyle behaviors. A higher number of health behaviors and factors at ideal levels is associated with significantly lower cardiovascular events.^3,4,5,6,7,8,9^ However, all of these studies were based on a single assessment of cardiovascular health status. An underexplored issue is whether the overall ideal cardiovascular health status over time (ie, trajectory) is associated with subsequent CVD risk as modified. Two studies reported that the improvement in CHS was positively associated with better cardiovascular function and structure markers.^10,11^ However, to our knowledge, no prospective data have been published to examine the direct associations between the CHS trajectory patterns over certain periods and subsequent CVD risk. Therefore, we investigated whether the trajectories of CHS over 4 years were associated with lower risks of developing CVD in a large, prospective cohort including 74 701 Chinese adults after controlling for potential confounders.

## Methods

### Study Population

The Kailuan study is a prospective cohort study conducted in the Kailuan community in Tangshan, Republic of China, which is a large, modern city southeast of Beijing. Detailed study design and procedures have been described in detail.^3,12,13,14,15,16^ Since June 2006, a total of 101 510 adult participants, including 81 110 men and 20 400 women, were enrolled from 11 hospitals in the Kailuan community and underwent questionnaire assessments, clinical examinations, and laboratory tests. All participants were then followed up every 2 years and the incidence of chronic diseases (eg, CVD) was recorded annually. In the present study, CHS trajectories were developed from 2006 to 2010 to predict CVD risk from 2010 to 2015. Data analysis was performed from January 1, 2006, to December 31, 2015.

The study protocol was approved by the ethics committee of the Kailuan General Hospital. All participants provided written informed consent; they did not receive financial compensation. This study followed the Strengthening the Reporting of Observational Studies in Epidemiology (STROBE) reporting guideline for cohort studies.

Included in the present analyses were 74 701 participants free of CVD and cancer in or before 2010 (ie, the baseline of present analyses). The flowchart of the participant selection process is shown in the eFigure in the Supplement. Compared with those who were not included in the present analysis owing to missing 2008 and 2010 CHSs, included participants had similar 2006 CHSs (mean, 8.7 vs 8.5 in 2006) but were older (mean age, 50.7 vs 49.0 years in 2006) and had a smaller proportion of men (77.9% vs 86.4%).

### Assessment of the CHS and Covariates

All participants had baseline and follow-up evaluations in the 11 hospitals of the Kailuan community. Information on demographic characteristics, medical comorbidities (eg, CVDs), home medications, and lifestyle factors, including smoking status, alcohol consumption, and physical activities were collected via standardized questionnaires. Owing to the lack of detailed dietary data during 2006-2010 and considering the influence of salt intake on CVD risks among the Chinese population, total salt intake derived from a questionnaire was used as a surrogate measure for diet quality, as described previously.^3,7,12^ Physical examinations (eg, weight, body mass index, height, blood pressure) were conducted by trained field workers (ie, physicians and nurses) during each survey. Laboratory tests, including total cholesterol, triglyceride, high-density lipoprotein cholesterol, low-density lipoprotein cholesterol, glucose, and high-sensitivity C-reactive protein levels, were assessed by an auto analyzer (Hitachi 747; Hitachi) at the central laboratory of Kailuan General Hospital.^3,7,12,13,14^

The CHS scoring system as previously described was used to quantify the overall cardiovascular health behaviors and factors.^2^ For each component of the 7 cardiovascular health metrics, the definitions of poor (0 points), intermediate (1 point), and ideal (2 points) were assigned, as documented in eTable 1 in the Supplement. The total score ranged from 0 (worst) to 14 (best).

In 2014, dietary data were collected with a semiquantitative, validated food-frequency questionnaire in 59 043 active participants of the Kailuan study.^17,18^ A healthy diet score (1 of the CHS components) was calculated based on the consumption of fruits, vegetables, fish, sodium, sweets, sugar-sweetened beverages, and whole grains,^2^ There was a strong association between higher perceived salt intake and lower healthy diet score (eTable 2 in the Supplement). The age- and sex-adjusted mean healthy diet scores were 1.87, 0.98, and 0.70, respectively, across the lowest, moderate, and the higher perceived salt intake groups (*P* value for trend <.001).

### Ascertainment of Incident CVD Events

The main outcomes were incident CVD events (including both myocardial infarction and stroke). The diagnosis of CVD was described previously.^13,15,16,19^ In brief, all participants were linked to the Municipal Social Insurance Institution and the Hospital Discharge Register for incidence of CVD, which cover all of the Kailuan study participants. To further identify potential CVD events, we reviewed the discharge lists from the 11 hospitals during 2006 to 2015 and asked for a history of CVD via a questionnaire during the biennial interview. For all suspected CVD events, 3 experienced physician adjudicators who were blinded to the study design reviewed the medical records. Incident myocardial infarction was diagnosed according to the World Health Organization’s Multinational Monitoring of Trends and Determinants in Cardiovascular Disease criteria on the basis of clinical symptoms and dynamic changes in cardiac enzymes and/or biomarker concentrations and electrocardiogram results.^13,15,20^ Stroke was diagnosed according to the World Health Organization criteria,^21^ based on signs, symptoms, neuroimages (from computed tomographic or magnetic resonance imaging), and other diagnostic reports, as detailed previously.^16,19^Mortality information was collected from provincial vital statistics offices.^19^ Study clinicians (S.W. and Y.W.) reviewed death certificates.

### Statistical Analysis

We computed the person-time of follow-up for each participant from the date of the 2010 survey (baseline) to the date of the CVD diagnosis, death, loss to follow-up (4678 of 74 701 [6.3%]), or the end of follow-up (December 31, 2015), whichever came first.

The CHSs in 2006, 2008, and 2010 were computed and the trajectories during 2006-2010 were then identified by latent mixture modeling, within the PROC TRAJ procedure, as detailed previously.^15,16,22^ Model fit was assessed using the bayesian information criterion and the number of participants in each trajectory (>5% of overall population).

Cox proportional hazards regression was used to calculate the hazard ratios (HRs) and 95% CIs of risk of developing CVD during 2010-2015 across the CHS trajectories and the proportional hazards assumption was satisfied. Because age and sex are strong determinates of exposure and outcomes, these factors were adjusted in model 1. Further adjustments were made for educational level (elementary school, high school, or college or above), occupation (coal miner, other blue-collar jobs, or other), income (>800 Chinese Yuan [$ 119] per month or ≤ 800 Chinese Yuan), alcohol consumption (never, past, current, <1 time per day or current, ≥1 times per day), and mean serum high-sensitivity C-reactive protein concentrations because these factors are known to be associated with both cardiovascular health metrics and CVD risk. To understand whether the potential association between the CHS trajectories and CVD could be modified by age, sex, and high-sensitivity C-reactive protein concentration, potential interactions were assessed between the CHS trajectories and these factors after controlling for the aforementioned covariates. Subgroup results, stratified by these factors, are presented.

Several sensitivity analyses were also conducted. To examine whether this association could be explained by a single CHS during the follow-up, we additionally adjusted for 2006 and 2010 CHSs. To reduce the possibility of reverse causality, lag-analysis was conducted by excluding incident CVD or death, separately, with onset during the first 2 years of follow-up. Because approximately one-third (n = 23 569) of the study participants worked in coal mines, we also conducted a sensitivity analysis after excluding these participants.

The association between the annual change rate of CHS during 2006-2010, as a secondary exposure, and CVD risk was examined. To explore the potential nonlinearity of this association, the continuous CHS annual change rate was used to fit a restricted cubic spline model with 4 knots, after adjustment for the effects of potential confounders and the 2006 CHS, one of the major determinants of change in CHS and CVD risk. Significance in trend was examined in the Cox proportional hazards regression models by using the annual change rate of CHS as a continuous variable. To examine whether this association was largely driven by any of the factors included in the CHS, we conducted an analysis by removing individual factors from the total score, 1 at a time, and recalculated the annual change rate of the new CHS.

All statistical tests were 2-sided, and *P* < .05 was regarded as significant. Statistical analyses were performed using SAS, version 9.3 (SAS Institute Inc).

## Results

**Figure 1.**
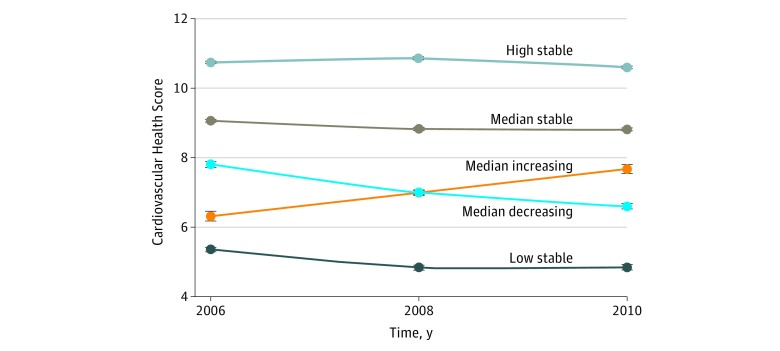
Mean Cardiovascular Health Score in 2006, 2008, and 2010, According to 5 Cardiovascular Health Score Trajectory Patterns The Cardiovascular Health Score varies from 0 to 14, with the highest score representing the lowest risk of cardiovascular disease. Error bars indicate 95% CI.

A total of 74 701 adults were included in the analyses. Mean (SD) age at baseline was 49.6 (11.8) years; 58 216 (77.9%) of the participants were men and 16 485 (22.1%) were women. Five distinct trajectory patterns were identified according to both baseline CHS and patterns over time (Figure 1): low-stable (n = 4393; mean CHS range, 4.6-5.2 during 2006-2010), moderate-increasing (n = 4643, mean CHS increased from 5.4 in 2006 to 7.8 in 2010), moderate-decreasing (n = 14 853, mean CHS decreased from 7.4 in 2006 to 6.3 in 2010), high-stable I (n = 36 352, mean CHS range, 8.8-9.0 during 2006-2010), and high-stable II (n = 14 461, mean CHS range, 10.9-11.0 during 2006-2010).

The mean CHS slightly declined from 2006 to 2010 (8.7 vs 8.4; *P* < .001). The basic characteristics of these individuals in 2006, according to CHS trajectories during 2006-2010, are presented in Table 1. During the follow-up period between 2010 and 2015, 1852 incident CVD events (1401 stroke cases, 429 myocardial infarction cases, and 22 stroke and myocardial infarction cases) were identified. The CHS trajectories were significantly associated with risk of CVD incidence (Table 2). Relative to the low-stable trajectory, the high-stable II trajectory was associated with lower subsequent risk of CVD (adjusted HR, 0.21; 95% CI, 0.16-0.26), after adjusting for potential confounders.

**Table 1.** Basic Characteristics of 74 701 Participants According to the Trajectories of CHS From 2006 to 2010^a^

Basic Characteristic	CHS Trajectory				
	Low-Stable	Moderate-Increasing	Moderate-Decreasing	High-Stable I	High-Stable II
No. of participants (%)	4393 (5.9)	4643 (6.2)	14 853 (19.9)	36 352 (48.7)	14 461 (19.4)
Age, mean (SD), y	49.0 (9.4)	50.7 (10.4)	50.2 (10.9)	50.5 (12.0)	46.3 (12.8)
Men, No. (%)	4201 (95.6)	4313 (92.9)	13 311 (89.6)	29 287 (80.6)	7104 (49.1)
CHS in 2006, mean (SD)^a^	5.18 (1.30)	5.44 (0.86)	7.74 (1.09)	9.03 (1.25)	10.98 (1.06)
hs-CRP, mg/L, median (IQR)	2.65 (2.00)	2.35 (1.82)	2.39 (1.81)	2.10 (1.60)	1.71 (1.26)
Educational level, No. (%)					
Elementary school	487 (11.1)	594 (12.8)	1437 (9.7)	3137 (8.6)	775 (5.4)
High school	3640 (82.9)	3831 (82.5)	12 593 (84.8)	30 984 (85.2)	11 663 (80.7)
College or above	261 (5.9)	211 (4.5)	817 (5.5)	2209 (6.1)	2017 (13.9)
Income >$119/mo, No. (%)	749 (17.0)	742 (16.0)	2187 (14.7)	4834 (13.3)	2309 (16.0)
Alcohol intake, No. (%)					
Never	1208 (27.5)	1475 (31.8)	7157 (48.2)	22 388 (61.6)	11 160 (77.2)
Past	172 (3.9)	216 (4.7)	562 (3.8)	11 27 (3.1)	254 (1.8)
Light^b^	275 (6.3)	344 (7.4)	961 (6.5)	2173 (6.0)	714 (4.9)
Moderate^b^	211 (4.8)	247 (5.3)	641 (4.3)	1463 (4.0)	416 (2.9)
Heavy^b^	1899 (43.2)	1688 (36.4)	3679 (24.8)	5307 (14.6)	759 (5.2)
Occupation, No. (%)					
Coal miner	2093 (47.7)	2258 (48.7)	5625 (38.0)	11 164 (30.8)	2429 (16.8)
Other blue-collar	2078 (47.4)	2163 (46.7)	8434 (56.9)	22 920 (63.2)	10 325 (71.6)
White-collar	214 (4.9)	211 (4.6)	760 (5.1)	2186 (6.0)	1667 (11.6)

Abbreviations: CHS, cardiovascular health score; hs-CRP, high-sensitivity C-reactive protein; IQR, interquartile range.

SI conversion factor: To convert hs-CRP to nanomoles per liter, multiply by 9.524.

^a^ The CHS varies between 0 to 14, with the highest score representing the lowest risk of cardiovascular disease.

^b^ Light drinker: 0.1 to 0.4 servings per day for women and 0.1 to 0.9 servings per day for men; moderate: 0.5 to 1.5 servings per day for women and 1 to 2 servings per day for men; and heavy: more than 1.5 servings per day for women and more than 2 servings per day for men; based on 15 g per serving of alcohol.

**Table 2.** Incidence of Cardiovascular Diseases According to Trajectories of CHS From 2006 to 2010^a^

Characteristic	CHS Trajectory, HR (95% CI)				
	Low-Stable	Moderate-Increasing	Moderate-Decreasing	High-Stable I	High-Stable II
Cardiovascular disease					
Cases, No.	230	177	537	785	123
Incidence rate, per 1000 person-years	9.96	7.18	6.78	4.00	1.57
Age- and sex-adjusted model	1 [Reference]	0.65 (0.54-0.80)	0.64 (0.55-0.75)	0.37 (0.32-0.43)	0.19 (0.16-0.24)
Multiple model 1^b^	1 [Reference]	0.67 (0.55-0.81)	0.64 (0.55-0.75)	0.38 (0.33-0.44)	0.21 (0.16-0.26)
Multiple model 2^c^	1 [Reference]	0.68 (0.56-0.83)	0.81 (0.67-0.97)	0.54 (0.43-0.66)	0.34 (0.25-0.47)
Multiple model 3^d^	1 [Reference]	0.94 (0.74-1.18)	0.77 (0.65-0.92)	0.60 (0.48-0.75)	0.40 (0.29-0.56)
Excluding coal miners^b^	1 [Reference]	0.63 (0.48-0.83)	0.64 (0.52-0.79)	0.40 (0.33-0.49)	0.20 (0.15-0.27)
2-y Lag analysis^b^	1 [Reference]	0.79 (0.63-1.00)	0.71 (0.58-0.85)	0.42 (0.35-0.50)	0.23 (0.17-0.30)
Stroke					
Cases, No.	180	143	406	605	89
Multiple model 1^b^	1 [Reference]	0.79 (0.63-1.00)	0.71 (0.58-0.85)	0.42 (0.35-0.50)	0.23 (0.17-0.30)
Myocardial infarction					
Cases, No.	51	36	138	191	35
Multiple model 1^b^	1 [Reference]	0.61 (0.40-0.94)	0.71 (0.51-0.98)	0.39 (0.28-0.53)	0.25 (0.16-0.40)

Abbreviations: CHS, cardiovascular health score; HR, hazard ratio.

^a^ The CHS varies between 0 to 14, with the highest score representing the lowest risk of cardiovascular disease.

^b^ Model 1 adjusted for age, sex educational level (elementary school, high school, or college or above), income level (income >800 Chinese Yuan [$119]/mo or income ≤800 Chinese Yuan/mo), occupation (coal miner, other blue-collar jobs, or other), alcohol consumption (never; past; current <1 time/d; or current, ≥1 times/d), and mean serum concentration of high-sensitivity C-reactive protein during 2006-2010 (quartile).

^c^ Model 2 adjusted as multiple model 1 plus CHS at 2006.

^d^ Model 3 adjusted as multiple model 2 plus CHS at 2010.

**Figure 2.**
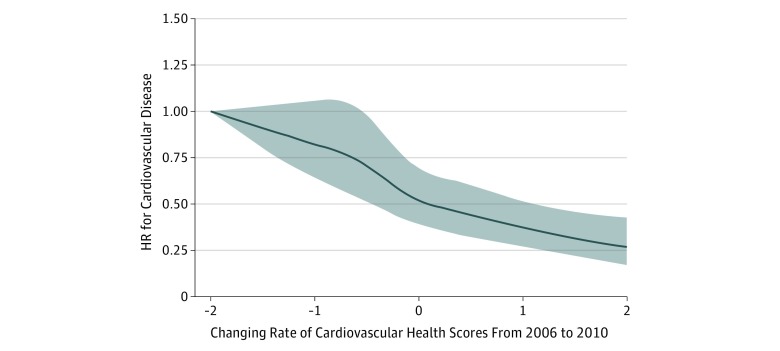
Adjusted Hazard Ratios (HRs) of Cardiovascular Disease Risk, According to Annual Change Rate of the Cardiovascular Health Scores During 2006-2010 Adjusted for age, sex, 2006 cardiovascular health score, educational level (elementary school, high school, or college or above), income level (>$119 per month or ≤$119 per month), occupation (coal miner, other blue-collar jobs, or other), alcohol consumption (never, past, current, <1 time per day or current, ≥1 times per day), and serum concentrations of high-sensitivity C-reactive protein. Data were fitted by a restricted cubic spline Cox proportional hazards regression model. The cardiovascular health score ranges from 0 to 14, with the highest score representing the lowest risk of cardiovascular disease. Shaded area indicates 95% CIs.

The results did not materially change after additional adjustment for the 2006 or 2010 CHS, excluding coal miners, or excluding incident CVD cases that occurred during the first 2 years of follow-up (Table 2). For example, the adjusted HR, comparing the high-stable II trajectory with the low-stable trajectory, was 0.34 (95% CI, 0.25-0.47) after adjusting for the 2006 CHS, 0.40 (95% CI, 0.29-0.56) after adjusting for the 2010 CHS, 0.20 (95% CI, 0.15-0.27) after excluding coal miners, and 0.23 (95% CI, 0.17-0.30) in the 2-year lag analysis. The associations between CHS trajectories and subsequent CVD risk appeared to be more pronounced among participants with younger age, relative to those 60 years or older at the baseline (*P* = .01 for interaction) (eTable 3 in the Supplement). We did not find significant interaction between the CHS trajectories and sex, and high sensitivity C-reactive protein concentrations (*P* > .05 for interaction for all) (eTable 3 in the Supplement). Consistently, baseline CHS status (eTable 4 in the Supplement) and improvement in CHS (ie, improvement in overall cardiovascular health status) from 2006 to 2010 (Figure 2) were associated with a significantly lower risk of CVD incidence (*P* for trend = .007). We obtained similar results when removing each CHS component from the total CHS (Table 3).

**Table 3.** Incidence of Cardiovascular Diseases According to Change Rate in CHS From 2006 to 2010, After Removing Individual Cardiovascular Health Components From the Total Score^a^

Characteristic	HR (95% CI) for 1-U Increase per Year^b^	*P* Value
Original CHS	0.89 (0.81-0.97)	.007
Removed component		
Smoking	0.88 (0.80-0.97)	.009
Salt intake	0.90 (0.82-0.98)	.02
Physical exercise	0.91 (0.83-1.00)	.04
Total cholesterol level	0.89 (0.81-0.97)	.01
Blood pressure	0.87 (0.79-0.95)	.003
Fasting blood glucose level	0.92 (0.84-1.01)	.07
Body mass index	0.86 (0.79-0.95)	.002

Abbreviation: CHS, cardiovascular health score.

^a^ The CHS varies between 0 to 14, with the highest score representing the lowest risk of cardiovascular disease.

^b^ Adjusted for age, sex, 2006 CHS, educational level (elementary school, high school, or college or above), income level (income >800 Chinese Yuan [$119]/mo or income ≤800 Chinese Yuan/mo), occupation (coal miner, other blue-collar jobs, or other), alcohol consumption (never; past; current, <1 time per day; or current, ≥1 times per day) and mean serum concentration of high-sensitivity C-reactive protein.

## Discussion

In this prospective study, 5 distinct CHS trajectories were identified and these patterns were associated with altered CVD risk. For example, participants who maintained the highest CHS (ie, the best overall cardiovascular health status) over 4 years (ie, the high-stable II trajectory) had a 79% lower risk of CVD incidence (ie, the adjusted HR of 0.21), relative to those with a consistently worst cardiovascular health status. We also observed a significant association between improvements in the participants’ overall cardiovascular health status by optimizing cardiovascular health behaviors and factors and lower subsequent risk of CVD, independent of baseline cardiovascular health status.

To our knowledge, this large-scale, community-based study is the first to suggest a potential association between cardiovascular health status over time and CVD risk. These observations add to the database suggesting that there is an inverse association between the numbers of ideal cardiovascular health metrics and a wide range of health outcomes, including myocardial infarction, stroke, heart failure, and CVD mortality across different ethnic groups.^3,4,5,8,9,23^ Health behaviors and factors have significant and substantial associations with higher longevity and CVD-free survival in both men and women.^8,24,25,26^ These findings are consistent with previous observations of an inverse association between baseline cardiovascular health metrics and the risks of stroke, CVD, and mortality^3,7,23^ Taken together, these data support the importance of strong public health efforts to improve cardiovascular health status among individuals.

We also observed a significant dose-response inverse association between the change in the CHS and subsequent risk of CVD, which was independent of baseline CHS status. Consistent with these data, in an evaluation of 6520 participants in the Atherosclerosis Risk in Communities study, greater improvements in the CHS from midlife to late life were significantly associated with lower CVD prevalence and better cardiovascular structure and function (eg, left ventricular structure, systolic and diastolic function, and biomarkers of myocardial stress and injury).^11^ In a Chinese cohort including 3951 participants, improvement in the CHS was inversely associated with the change of brachial-ankle pulse wave velocity and atherosclerosis progression.^10^ These observations suggest potential biological mechanisms underlying the observed association between change in the CHS and future CVD morbidity and mortality risk. Globally, the prevalence of individuals with ideal cardiovascular health scores, defined as meeting all 7 components, is low in the general population, estimated to be approximately 1% to 2% in adults.^5,6,7,8,27,28^ Determining whether and how much of an improvement in cardiovascular health metrics, which can be measured quantitatively by the CHS,^2^ is associated with a decreased risk of CVD would contribute to efforts to increase awareness and adherence to current risk reduction recommendations.

Although CHS trajectories were associated with an altered risk of CVD in both younger and older participants, overall effect sizes were slightly larger for the younger group. This age difference was consistent with previous studies of cardiovascular health metrics and CVD mortality^5^ and cognitive function.^29^ The findings highlight that improving overall cardiovascular health status in middle-aged adults is important to prevent the initial development of CVD risk factors.

### Strengths and Limitations

A strength of our study is that we repeatedly collected the information on the CHS, which may reduce random errors. Examining the CHS trajectories over a 4-year period may provide insights into the potential association between long-term, overall cardiovascular health and disease risk. Furthermore, the homogeneous nature of our cohort could reduce potential confounding factors due to racial/ethnic and health care disparities.

A potential limitation of the study is that our cohort only included Chinese adults from the Kailuan community. Hence, findings may not be generalizable to other populations. However, similar inverse associations between the numbers of ideal cardiovascular health metrics and CVD risks have been shown in multiple racial/ethnic groups, suggesting the broad generalizable nature of the data.^3,4,5,9^ Another limitation is that daily salt intake was used as a surrogate for diet quality because data regarding detailed dietary components were not available until 2014. A healthy diet score should be calculated on the basis of consumption of fruits, vegetables, fish, sodium, sweets, sugar-sweetened beverages, and whole grains.^2^ In a typical Chinese diet, excessive salt intake is common, estimated to average 9.1 g/d in urban areas and 11.5 g/d in rural areas.^30^ Given that salt intake was consistently found to be associated with higher CVD risk^31^ and excessive salt intake is a problem in China, salt intake was used as a surrogate for diet quality. In support of this decision, subsequent work (as described in Assessment of the CHS and Covariates of the Methods section herein) identified a strong association between salt intake and the healthy diet score in the Kailuan study. Furthermore, removing the diet score based on salt intake did not materially change the conclusions. Nevertheless, the result should be interpreted with caution, especially in populations with different dietary patterns. Physical activity was assessed using an invalidated questionnaire. However, previous studies based on the same population suggested an inverse association between physical activity levels and the risk of developing stroke,^3^ which provides indirect evidence regarding the questionnaire’s validity. The trajectory patterns identified by the latent mixture models are a posteriori and we failed to identify a low-increasing or a high-decreasing CHS pattern in the studied population.

## Conclusions

The results of this study suggest that CHS trajectories and an improvement in overall cardiovascular health status, as suggested by an increased CHS over time, might be associated with risk of CVD morbidity in a large Chinese cohort. These results suggest that implementing aggressive primordial preventive strategies to optimize cardiovascular health behaviors and risk factors will improve cardiovascular health. Further studies, particularly those with detailed data on diet and physical activity, are warranted to replicate our observations.

## Notes

Supplement. **eTable 1.** Definitions of Poor (0 Point), Intermediate (1 Point), and Ideal (2 Points) for Each Cardiovascular Health Component **eTable 2.** The Means and Standard Errors of the Healthy Diet Score, According to Salt Intake **eTable 3.** Hazard Ratios and 95% Confidence Intervals of Cardiovascular Diseases According to Trajectories of Cardiovascular Health Scores From 2006 to 2010, Stratified by Sex, Age, and High Sensitive C-Reactive Protein Concentration **eTable 4.** Hazard Ratios and 95% Confidence Intervals of Cardiovascular Diseases According to the Quintile of Cardiovascular Health Scores in 2010 **eFigure.** Flow Chart of Subject Selection Process
