# Supplementary material for: Association of Trajectory of Cardiovascular Health Score and Incident Cardiovascular Disease
Source: JAMA Netw Open. 2019 May 31;2(5):e194758. doi: 10.1001/jamanetworkopen.2019.4758 (PMC6547110; doi:10.1001/jamanetworkopen.2019.4758)
Supplement: Supplement. — eTable 1. Definitions of Poor (0 Point), Intermediate (1 Point), and Ideal (2 Points) for Each Cardiovascular Health Component eTable 2. The Means and Standard Errors of the Healthy Diet Score, According to Salt Intake eTable 3. Hazard Ratios and 95% Confidence Intervals of Cardiovascular Diseases According to Trajectories of Cardiovascular Health Scores From 2006 to 2010, Stratified by Sex, Age, and High Sensitive C-Reactive Protein Concentration eTable 4. Hazard Ratios and 95% Confidence Intervals of Cardiovascular Diseases According to the Quintile of Cardiovascular Health Scores in 2010 eFigure. Flow Chart of Subject Selection Process [file jamanetwopen-2-e194758-s001.pdf]

Supplementary Online Content

Wu S, An S, Li W, et al. Association of trajectory of cardiovascular health score and incident cardiovascular disease. *JAMA Netw Open*. 2019;2(5):e194758. doi:10.1001/jamanetworkopen.2019.4758

eTable 1. Definitions of Poor (0 Point), Intermediate (1 Point), and Ideal (2 Points) for Each Cardiovascular Health Component

eTable 2. The Means and Standard Errors of the Healthy Diet Score, According to Salt Intake

eTable 3. Hazard Ratios and 95% Confidence Intervals of Cardiovascular Diseases According to Trajectories of Cardiovascular Health Scores From 2006 to 2010, Stratified by Sex, Age, and High Sensitive C-Reactive Protein Concentration

eTable 4. Hazard Ratios and 95% Confidence Intervals of Cardiovascular Diseases According to the Quintile of Cardiovascular Health Scores in 2010

eFigure. Flow Chart of Subject Selection Process

This supplementary material has been provided by the authors to give readers additional information about their work.

© 2019 Wu S et al. *JAMA Network Open.*

eTable 1. Definitions of Poor (0 Point), Intermediate (1 Point), and Ideal (2 Points) for Each Cardiovascular Health Component

	Poor (0 point)	Intermediate (1 point)	Ideal (2 points)
Cardiovascular Health Behaviors			
Cigarette Smoking	Current smoker	Past smoker*	Never
Body Mass Index (BMI) (kg/m^2)	≥ 30	25-30	< 25
Diet , based on daily salt intake (g/d)	≥ 10	6-9	< 6
Physical Activity (moderate or vigorous exercise)	No physical activity	physical activity (20+ minutes per time) 1-2 times per week, during leisure time	physical activity (20+ minutes per time) 3 times per week, during leisure time
Cardiovascular Health Factors			
Cholesterol	Total cholesterol ≥ 240 or treated total cholesterol > 200 mg/dL	Total cholesterol 200-239 or treated total cholesterol < 200 mg/dL	Total cholesterol < 200 mg/dL
Glucose / DM	FPG ≥ 126 mg/dL or diagnosed DM with HbA_{1C} ≥ 7%	FPG 100-125 mg/dL or diagnosed DM with HbA_{1C} < 7%	FPG < 100 mg/dL
BP	Treated BP > 140/>90 and SBP ≥ 140 or DBP ≥ 90 mmHg	SBP 120-139 or DBP 80-89 or treated BP <140/<90 mmHg	BP <120/<80 mmHg

Abbreviations: DM, diabetes mellitus; FPG, fasting plasma glucose; HbA_{1C}, hemoglobin A_{1C}; BP, blood pressure; SBP, systolic blood pressure; DBP, diastolic blood pressure

*Past smoker was defined when a participant who reported to smoke previously but quit smoking already during the survey.

© 2019 Wu S et al. *JAMA Network Open.*

eTable 2. The Means and Standard Errors of the Healthy Diet Score*, According to Salt Intake

	<6 gram/day	6–9 gram/day	≧10 gram/day
n	6911	47475	4657
Crude	1.94±0.01	1.02±0.00	0.80±0.01
Age- and sex-adjusted	1.87±0.01	0.98±0.00	0.70±0.02
Age- and sex-adjusted mean, men	1.92±0.01	1.04±0.01	0.75±0.02
Age- and sex-adjusted mean, women	1.85±0.03	0.92±0.01	0.70±0.04

*The healthy diet score was calculated based on consumption of fruit/vegetable, whole grain, fish, salt and sugar-sweetened beverage

© 2019 Wu S et al. *JAMA Network Open.*

eTable 3. Hazard Ratios (HRs) * and 95% Confidence Intervals (95% CIs) of Cardiovascular Diseases According to Trajectories of Cardiovascular Health Scores From 2006 to 2010, Stratified by Sex, Age, and High Sensitive C-Reactive Protein (hs-CRP) Concentration

	**Low-stable**	**Moderate-increasing**	**Moderate-decreasing**	**High-stable I**	**High-stable II**	**p for interaction**
**Cardiovascular diseases**						
**Men**	1.00	0.57(0.24-1.34)	0.49(0.24-0.98)	0.31(0.16-0.60)	0.13(0.06-0.28)	0.58
**women**	1.00	0.67(0.55-0.82)	0.65(0.56-0.77)	0.38(0.33-0.45)	0.22(0.18-0.29)	
**age < 60 y**	1.00	0.66(0.52-0.82)	0.63(0.53-0.75)	0.36(0.30-0.43)	0.16(0.12-0.22)	0.001
**age≥ 60 y**	1.00	0.73(0.48-1.11)	0.73(0.51-1.03)	0.49(0.35-0.68)	0.36(0.24-0.54)	
**hs-CRP <3 mg/L**	1.00	0.78(0.57-1.06)	0.71(0.55-0.92)	0.41(0.32-0.53)	0.22(0.16-0.31)	0.60
**hs-CRP ≥3 mg/L**	1.00	0.61(0.47-0.79)	0.62(0.51-0.76)	0.37(0.30-0.45)	0.20(0.14-0.27)	

* Adjusted for age (ys), sex, education level (elementary school, high school or college or above), income level (income>800 Chinese Yuan/month or income≤800 Chinese Yuan /month), occupation (coal miner or other), alcohol consumption (never, past, current, <1times/d or current, 1+times/d) and C-reactive protein in 2006 (Quartile).

© 2019 Wu S et al. *JAMA Network Open.*

eTable 4. Hazard Ratios (HRs) and 95% Confidence Intervals (95% CIs) of Cardiovascular Diseases According to the Quintile of Cardiovascular Health Scores in 2010

	Q1	Q2	Q3	Q4	Q5	P for trend
**Cardiovascular disease**						
**Case**	581	339	652	166	114	
**Incidence rate, per 1000 person-year**	8.13	6.05	4.54	2.57	1.72	
**Multiple adjusted HR***	1.00	0.74(0.65-0.85)	0.57(0.50-0.63)	0.34(0.29-0.41)	0.29(0.24-0.36)	<0.001

*Adjusted for age (ys), sex, education level (elementary school, high school or college or above), income level (income>800 Chinese Yuan/month or income≤800 Chinese Yuan /month), occupation (coal miner or other), alcohol consumption (never, past, current, <1times/d or current, 1+times/d) and C-reactive protein in 2006 (Quartile).

© 2019 Wu S et al. *JAMA Network Open.*

eFigure. Flow Chart of Subject Selection Process

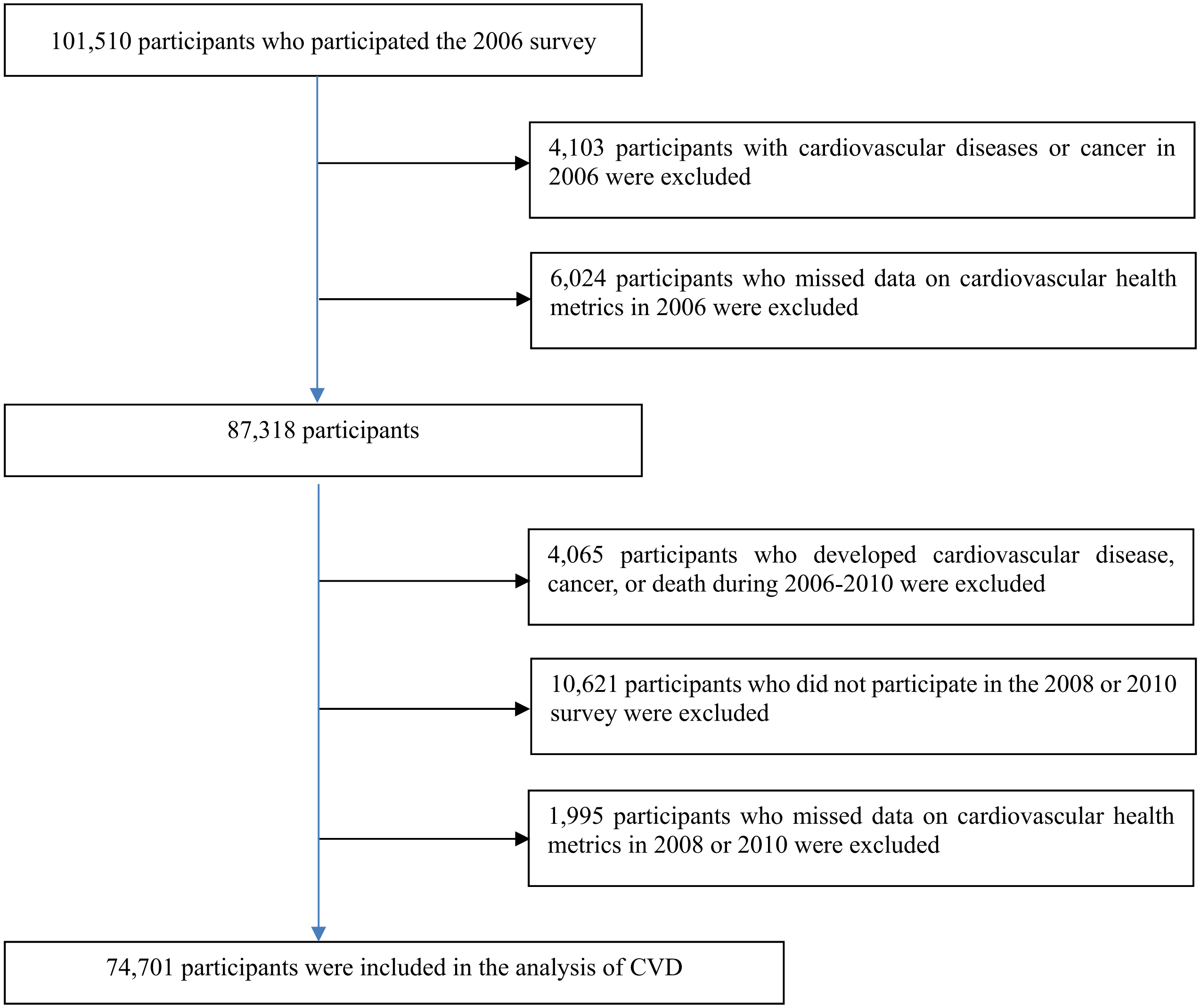

© 2019 Wu S et al. *JAMA Network Open.*
